# Characteristics of cardiac device infections in the Isala Hospital; a large volume tertiary care cardiology centre

**DOI:** 10.1007/s12471-015-0799-x

**Published:** 2016-01-11

**Authors:** R. Steenmeijer, A. Adiyaman, F. Demirel, H. C. F. Schram, J. J. J. Smit, P. P. H. M. Delnoy, A. R. Ramdat Misier, A. Elvan

**Affiliations:** Department of Cardiology, Isala Hospital, Dr. Van Heesweg 2, 8025 AB Zwolle, The Netherlands

**Keywords:** Cardiac device infection, Staphylococcus aureus, Device extraction

## Abstract

**Aims:**

To determine the frequency, characteristics and risk factors of cardiac device infections in the Isala Hospital.

**Methods:**

We retrospectively studied all patients who underwent cardiac device procedures performed in the cardiac catheterisation lab and the operating room from 2010 to 2012. All patients who developed a cardiac device infection were reviewed for its characteristics.

**Results:**

31/2026 patients developed a cardiac device infection (1.5 %). One (3.2 %) patient died within 30 days of hospitalisation. Device infection rates for procedures in the catheterisation lab and operating room were similar (*p* = 0.60). Positive cultures were present in 27/31 (87 %) cases. These consisted predominantly of micro-organisms that are part of the skin flora (84 %). The mean time between device procedure and infection was 14 ± 21 months (range 0–79). Cardiac device infection was significantly associated with device revision, (65 % were revisions in patients with device infection vs. 30 % revisions in patients without device infection, *p* = 0.011) and placement of a left ventricular lead in pacemaker implantations (59 % of patients with vs. 51 % of patients without device infection, *p* < 0.001).

**Conclusion:**

The frequency of cardiac device infection was 1.5 % with a mortality of 3.2 % within 30 days, which is lower compared with other registries. Cardiac device infections were associated with device revisions and placement of left ventricular leads in pacemaker implantations.

## Introduction

The relative number of cardiac device infections is rising together with an increase in device implantations [1–3]. Cardiac device infection is associated with substantial morbidity and mortality [1, 2]. Risk factors associated with device infections are the number of previous device procedures, renal insufficiency, post-procedural haematoma, fever < 24 h after the procedure, and complexity and duration of the procedure [1, 4, 5].

The Isala is a large volume tertiary care heart centre, with advanced experience in the management of cardiac device complications, including infections. We previously published on lead dysfunction rates in our hospital [6]. Due to the rising number of cardiac device infections, a nationwide program was initiated from the National Society of Cardiology (NVVC) in the Netherlands in 2008 in order to reduce the incidence along with its morbidity and mortality rates. Especially for implantable cardioverter defibrillators (ICDs), infection and complication rates were registered. At present, limited data are available regarding complications of cardiac device implantations in the Netherlands. Our main goal, therefore, was to examine the frequency, characteristics and possible risk factors for cardiac device infections in our large volume centre.

## Methods

Standardly, device procedures were performed in the cathlab; however, when submuscular device implantations were planned, or procedures were under general anaesthesia, the operating room was used. All patients who underwent cardiac device procedures were treated with prophylactic antibiotics (cefazolin 1 gram intravenously) 1 h before and 3 h after the procedure. At the discretion of the operator, antibiotic treatment was continued with oral clindamycin (mostly in device revisions). We reviewed all cases of cardiac device infection between 1 January 2010 and 31 December 2011. Cases were identified from a retrospectively studied prospective registry including all patients who underwent a cardiac device procedure in the Isala in Zwolle, the Netherlands. There were no exclusion criteria. Independent research assistants registered pre-defined data in an electronic research form. These data included age, sex, known risk factors for cardiac device infections such renal insufficiency, number of leads, type of device, time after device procedure and number of device procedures. Patients were followed according to the standard routine at the outpatient clinic, 6–8 weeks after device procedure, and at 6-month intervals thereafter, or more frequently when deemed necessary. Generally, cardiac resynchronisation therapy and ICDs were monitored in the Isala, and not in the referring centre. If controls were performed in a referral centre, we obtained information regarding the patient from that referring centre. A thorough screening of the medical files was performed in all cases. The diagnosis of cardiac device infection was based on the combination of suspected clinical history, signs of pocket infection on physical examination, and with transthoracic and/or transoesophageal echocardiography where appropriate. A second physician specialised in cardiac device implantations (e.g. a cardiac electrophysiologist) validated the diagnosis. In all cases blood cultures were taken. When a device infection was present, e.g. a pocket infection or device-related endocarditis, the standard treatment was to remove all device material and aggressively treat the patient with culture-guided intravenous antibiotics for a period of 2–6 weeks (depending on the type of infection). Lead extraction was performed using a standard stepwise approach in all patients. After leads were dissected free from the scar tissue in the pocket, the anchor sleeves were removed and the active fixation mechanism was retracted. After that, controlled manual traction was attempted. If the lead could not be easily removed, then an appropriately sized locking stylet (Liberator Universal locking stylet, Cook Vascular, USA) was placed, and a silk suture was tied around the lead to bind the insulation to the conductors and to keep the insulation from bunching in front of the sheath. Controlled manual traction was again attempted with the locking stylet in place, making sure not to disrupt the lead integrity. If still unsuccessful a hand-powered mechanical rotational dilator Evolution sheath was used. All patients requiring this sheath underwent the procedure under general anaesthesia supervised by a cardiac anaesthesiologist, and with continuous transoesophageal echocardiographic monitoring. We did not use Laser when requiring a powered extraction tool. When lead vegetations with a magnitude of > 3 cm or valvular endocarditis were present, the open chest procedure was performed with the cardiothoracic surgeon in the operating room. Cultures were systematically obtained from the blood and the device pocket during extraction. If re-implantation of a new device was performed, this took place on the contralateral side and at least one week after the last negative blood culture. Pacing-dependent patients received a temporary pacemaker via the contralateral jugular vein when a percutaneous lead extraction was performed, or epicardial leads when surgery had to be performed to remove all device-related material.

### Statistical analysis

Continuous variables are reported as mean ± standard deviation. Categorical variables are reported as number and percentage. Normality of distribution was tested by the Kolmogorov-Smirnov test or distribution plots. Statistical significance between differences was calculated by Chi-squared test, Fisher’s exact test, Mann-Whitney or student-T-test where appropriate. *P*-values (two-sided) ≤ 0.05 were considered statistically significant. Statistical analysis was performed using SPSS v17.0 (SPSS Inc, Chicago).

## Results

During the study period, 2026 device-related procedures (excluding device infection-related procedures) were performed in 1867 patients. Of these procedures, 1702 (84 %) took place in the cardiac catheterisation lab used specifically for cardiac electrophysiological procedures and 324 (16 %) in the operating room. There were 1180 primary implantations and 846 revisions (mostly generator replacement). Device replacement was performed in 771 procedures and there were 75 lead revisions without device replacement. The proportion of revisions on the operating room was 30 % for pacemakers and 34 % for ICDs, whereas this was 23 % for pacemakers and 14 % for ICDs in the catheterisation lab.

**Fig. 1:**
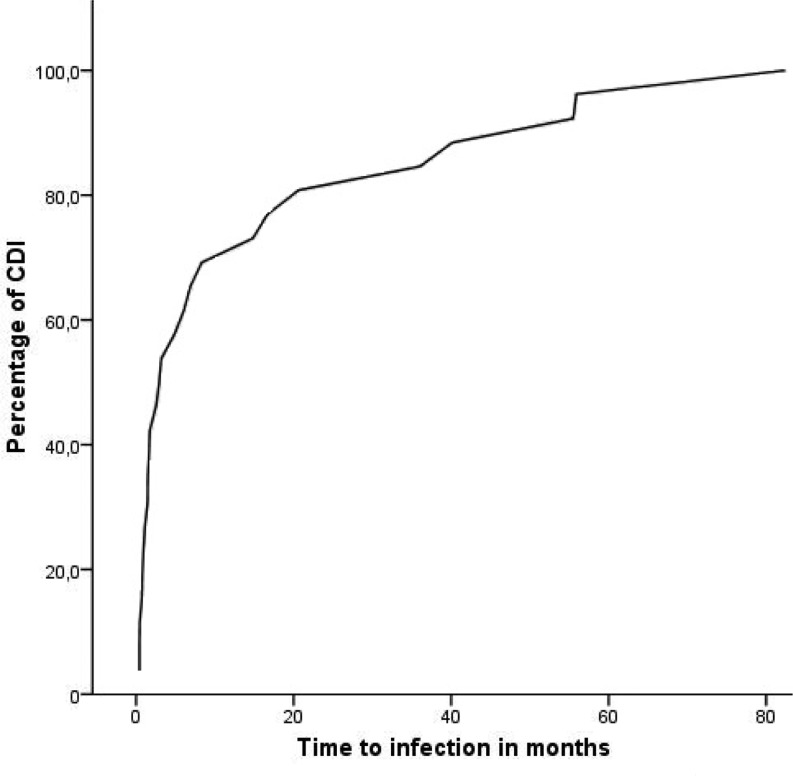
Overview of cumulative cardiac device infections with time after procedure (months) on the X-axis and number of infections (in percentage of total) on the Y-axis

In 31 of 2026 procedures (1.5 %), a cardiac device infection occurred during follow-up. Of these 31 cases, 13 had a primary implantation in secondary care centres in the area. There were no patients lost to follow-up. The mean time between device procedure and device infections was 14 ± 21 months (0–79 months). Clinical characteristics of patients with a device infection are presented in Table 1. In 6 cases (19 %) a device infection occurred within one month after the primary implantation or revision and in 8 cases (26 %) the device infection was more than 1 year after the last device procedure. Thus, about 60 % of the device infections occurred in the first year (Fig. 1).

**Table 1:** Characteristics of patients with a cardiac device infection

**Age**	69 ± 18 (years)
**% Male**	61 % (19)
**BMI**	26 ± 4 (kg/m^2^)
**Diabetes mellitus**	5 (16 %)
**Renal failure** ^a^	1 (3,2 %)
**Mean creatinine**	121 ± 19 (μmol/L)
**Malignancy**	3 (10 %)
**Primary implantation**	11 (35 %)
**Revision**	20 (65 %)
*Device revision*	14 (70 %)
*Lead revision*	4 (20 %)
*Upgrade*	2 (10 %)
**Pacemaker**	17 (55 %)
*1–2 leads*	13 (76 %)
*2* + *leads*	4 (24 %)
**ICD**	14 (45 %)
*1–2 leads*	11 (79 %)
*2* + *leads*	3 (21 %)
**Last procedure in OR**	3 (10 %)
**Last procedure in CCL**	28 (90 %)
**Time after revision**	323 days (13-1677)
**Time after implantation**	655 days (14-2472)
**Vitamin K antagonist**	15 (48 %)
**Corticosteroid use**	0 (0 %)

*BMI* body mass index, *ICD* implantable cardioverter defibrillator, *OR* operating room, *CCL* cardiac catheterisation lab.

^a^The eGFR was calculated by the MDRD formula. Renal failure was defined as an eGFR < 30.

All patients with cardiac device infections underwent extraction of all device-related material. Only a pocket infection was present in 24 of 31 these patients. In 7 of the 31 cases (23 %), besides a pocket infection, lead endocarditis with vegetations was present. In one case there was involvement of the tricuspid valve, which was successfully treated conservatively with antibiotics. All cases of cardiac device infections were treated percutaneously in the operating room. In 8 cases a temporary lead was needed. One patient developed vegetations in the right atrium, after a percutaneous extraction of the whole system, and these vegetations had to be removed surgically. One patient died within 30 days (3.2 % mortality in patients with cardiac device infections and 0.05 % mortality compared with the whole study cohort); unfortunately the cause of death could not be identified. When follow-up was extended to 1 year, 1 additional patient died, however not due to a device infection or its consequences. The 1-year mortality was not significantly different (*p* = 0.92) compared with the control group (112/1867, 6.0 %). No difference was present in the frequency of cardiac device infections between procedures performed in the cardiac catheterisation lab or the operating room (*p* = 0.60).

### Aetiology of cardiac device infection

**Fig. 2:**
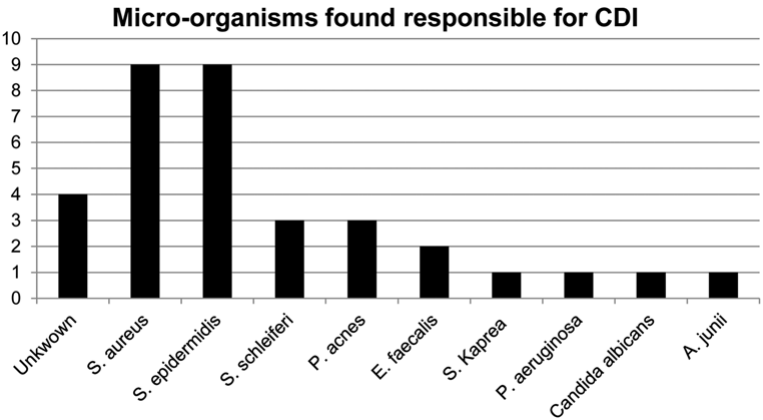
Micro-organisms found responsible for cardiac device infections

In all 31 cases multiple blood, device and lead cultures were taken. In 87 % of the cases (27/31) cultures were positive. In 12/27 cases (44 %) blood cultures were positive, in 67 % (18/27) a micro-organism was isolated from the pocket/wound and in 48 % micro-organisms were found at the extracted leads. These consisted predominantly of micro-organisms that are part of the skin flora (84 %). *Staphylococcus aureus* and *Staphylococcus epidermidis* were the most frequently found pathogens of device infection in this study. An overview of rates of device infection pathogens is presented in Fig. 2.

When we compare the characteristics of cases of cardiac device infections with all cardiac device procedures, device revision was significantly associated with device infection (65 % of patients with a device infection had a revision vs. 30 % of patients without infection, *p* = 0.01). No difference was observed, however, for time to device infection between device revisions and primary implantations (323 days for revision vs. 655 days for primary implantation, *p* = 0.15). Further, placement of a left ventricular lead in pacemaker implantations was associated with device infection (59 % of patients with vs. 51 % of patients without infection, *p* < 0.001). All other characteristics described in Table 2 were not significantly different.

**Table 2:** Comparison between case of cardiac device infection (CDI) and all cases

	CDI	All cases (minus CDI)	*p*-value
**Age**	69 ± 19	67 ± 13	*p* = 0.667
**% Male**	19/31	1259/1867	*p* = 0.469
**Diabetes mellitus**	5/31	313/1867	*p* = 0.925
**Renal failure**	1/31	42/1744	*p* = 0.769
**Device revision**	20/31	846/2026	*p* = 0.011
**Pacemaker**	17/31	468/1180	*p* = 0.089
**3 leads**	6/17	24/468	*p* < 0.0001
**ICD**	14/31	692/1180	*p* = 0.089
**3 leads**	3/14	233/692	*p* = 0.336
**Vitamin K antagonist**	15/31	689/1867	*p* = 0.189
**All-cause mortality**	2/31	112/1867	*p* = 0.916

## Discussion

Our present study shows that the incidence of cardiac device infection in our hospital is 1.5 %, and is mainly caused by skin-related bacteria. Risk factors associated with device infection are device revision (e.g. generator change) and number of pacemaker leads.

The reported rate of cardiac device infection throughout the world ranges between 0.8–5.8 % [7–9]. At 1.5 %, the reported frequency in our hospital is relatively low. Possibly, the aggressive protocol for periprocedural prophylactic antibiotics in patients at high risk for device infection (mainly in patients with device revisions with additional lead placement, or when procedure durations were longer than 3 h) could have contributed to this. In general, the high level of experience of operating physicians is associated with lower rates of infection. The true infection rate might be even lower, because 13/31 patients had a primary implantation in the referring hospital.

The reported 30-day mortality rate in our study was as low as 3.2 % and 1 year all-cause mortality rates were similar to patients without device infection. Again, mortality rates were lower in our present report, in comparison with other reports, which had mortality rates of 7.4–23 % [3, 10–12]. In these reports, however, only 8–41 % of patients underwent complete removal of the device and leads [11, 12]. Thus, our lower mortality rate could be caused by our aggressive approach of fast and complete system removal, preferably percutaneously, with aggressive and prolonged antibiotic treatment. The current European Society of Cardiology guidelines support this practice [13].

Risk factors for device infection were device revision and left ventricular lead placement. In our present report, this was the case in pacemakers but not in ICDs. We had expected that in both groups, a higher number of leads would be related to device infection, mainly because of the procedure duration. Differences in clinical characteristics of pacemaker versus ICD patients could be of influence in explaining this unexpected finding in our present report. Unfortunately, we could not perform multivariate analysis correcting for confounders, due to the low number of endpoints. The exact relation can therefore not be extracted from our database, and to refrain from hypothetical discussion, we decided not to speculate on this. Most previous reports indicate that a higher number of previous device procedures is one of the most important risk factors for device infection. Additionally, procedure time, pocket haematoma, procedure duration, renal dysfunction, low body mass index, diabetes and steroid use have been reported as risk factors [5, 14, 15]. It is at present unknown if prolonged use of prophylactic antibiotics in patients at high risk for device infection reduces the rate of device infection. Recently, an antibacterial envelope placed around the device generator in the pocket was developed, possibly further reducing infection rates [16].

In our present study, no significant difference was seen in cardiac device infection rate between device procedures that were performed in the catheterisation lab and the operating room. This is supported by the observations by Sohail et al. [15]. Possibly, this can be explained by the fact that most device infections are caused by skin-related micro-organisms, and could be introduced during the postoperative period, or thereafter. Most device infections were caused by *Staphylococcus* species commonly found on the human skin. Caution is needed with interpretation of the device and lead cultures, since there is a chance of contamination which might have led to false-positive cultures. There was an early infection rate (e.g. <1 month) of 23 %. It is likely that early infection is caused by primary contamination during the device procedure or in the directly following period. For the device infections that occur in a later time frame, it is not always easy to determine the cause of the infection. In one patient, in this study, device infection occurred after elective surgery for varicose veins. Iatrogenic causes of infection play a role in device infection, but it would be hard to lower the rate of this since it is common practice to give patients prophylactic antibiotics for most surgical procedures.

### Limitations

A major limitation is that we retrospectively analysed our database from a single centre. Since patients were followed once per 6 months, and we did not actively search for possible device infections between the regular follow-up visits, we could have missed possible patients with a device infection with symptoms not severe enough for the patient to attend to the hospital. Not all clinical variables were available for analysis (e.g. duration of the procedure and incidence of pocket haematoma), precluding the possibility to find associations of these variables with device infection. Another limitation is the fact that we compared two groups that were possibly not comparable at baseline due to the retrospective design of the study.

## Conclusion

We report relatively low numbers of device infection incidence, and low mortality rates when patients are prophylactically treated with antibiotics and, in case of an infection, treated aggressively. Risk factors for device infection were device revision and placement of left ventricular leads, and were mainly caused by skin-related bacteria.

### Funding

None.

### Conflict of interest

None declared.
